# Collection of patient-reported outcomes; - text messages on mobile phones provide valid scores and high response rates

**DOI:** 10.1186/1471-2288-14-52

**Published:** 2014-04-16

**Authors:** Anne Christie, Hanne Dagfinrud, Øystein Dale, Trenton Schulz, Kåre Birger Hagen

**Affiliations:** 1 National resource center for rehabilitation in rheumatology – NKRR, Department of Rheumatology, Diakonhjemmet Hospital, PO Box 23, Vinderen, 0319 Oslo, Norway; 2 Norwegian Computing Centre, PO Box 114, Blindern, 0314 Oslo, Norway

**Keywords:** Frequent data collection, Patient reported outcome, Rheumatic disease, Text-messages, Momentary assessment, Mobile phone, Paper questionnaires, Real-time assessment

## Abstract

**Background:**

Patients with inflammatory rheumatic diseases have expressed a need for more frequent measurement of relevant outcomes, due to the variations in their symptoms during the day and from day to day. At present, patient-reported outcomes are extensively collected with questionnaires completed with pen and paper. However, as a measurement tool in frequent data collection the questionnaires are impractical. In contrast, text messages on mobile phones are suitable for frequent data collection.

The aim of this study was two-fold; to compare daily registrations of patient-reported outcomes assessed with text-messages on mobile phones (SMS) or with questionnaires completed with pen and paper (P&P), with regard to scores and variation of scores, and to examine feasibility of the SMS method in a multicentre clinical study.

**Methods:**

To compare scores, 21 patients with an inflammatory, rheumatic disease performed daily assessments of pain, fatigue, stiffness and ability to carry out daily activities on a numeric rating scale (NRS). The patients were asked to assess the variables every other day with the SMS method and every other day with the P&P method for 28 consecutive days. In total each participant had to answer 14 P&P forms and 14 SMS messages. Mean scores and variation, expressed as the pooled standard deviation or as the average range between the maximum and minimum scores for the two methods, were compared using paired sample t-tests or Wilcoxon Signed Rank Test. To examine feasibility, 36 patients with an inflammatory, rheumatic disease assessed the same four variables with SMS twice a week for 35 weeks. Feasibility of the SMS method was expressed as mean response-rate (%) in the total group and per centre.

**Results:**

Mean scores, standard deviation of mean scores and mean range scores did not differ significantly between the two methods (p > 0.05). Response-rate with the SMS method was 97.9% for the whole group and for the three centres 97.1%, 98.3% and 98.4%, respectively.

**Conclusion:**

Outcomes assessed on numeric rating scales and reported with text-messages on mobile phones or with questionnaires completed with pen and paper provide comparable scores. Further, the SMS method provided high response rates (> 97%) in a multicentre setting. Our results encourage the use of text messages on mobile phones in studies requiring frequent data collection and real-time assessment, as in fluctuating diseases such inflammatory, rheumatic diseases.

## Background

Inflammatory rheumatic joint diseases, such as rheumatoid arthritis (RA) and ankylosing spondylitis (AS) are chronic and often disabling diseases causing pain, fatigue and functional limitations. Symptoms can vary both during the day and over time, even in patients with a stable disease [1]. In fact, the daily variation in pain, fatigue and global disease activity has been shown to be substantial [2].

Patient-reported health status is considered a key element in the assessment of rheumatic diseases and is part of the recommended core measures in clinical studies [3]. Patients have expressed a need for more frequent measurement of relevant outcomes, due to the variations in symptoms during the day and from day to day [4,5]. In rheumatologic practice and research patient-reported outcomes (PROs) are extensively collected with questionnaires completed with pen and paper (P&P). However, as a measurement tool in frequent data collection the P&P method is impractical and partly unreliable as the registrations are not always filled in at the intended time [6]. In contrast, text messages on mobile phones (SMS) are suitable for frequent data collection. Further, the messages are time stamped and thus real-time assessments, a feature precluding retrospective data reporting.

In a review addressing whether computer-administered methods were comparable to their P&P forms, 12 studies within rheumatology were identified [7]. All studies concluded that scores obtained via computer-administered methods and P&P forms were comparable, but none of the studies applied the SMS as a data collection method. To our knowledge, the SMS format has not been compared to the established method of P&P forms in frequent data collection or been evaluated for feasibility in clinical studies in patients with inflammatory rheumatic diseases.

### The aim of this study was two-fold

1. To compare daily registrations of patient-reported outcomes assessed with text-messages on mobile phones (SMS) or with pen and paper (P&P), with regard to scores and variation of scores.

2. To examine feasibility of the SMS method in a multicentre clinical study.

## Methods

Twenty-eight patients were included for comparisons of methods, and 41 patients, participating in a clinical multicentre study, were included for examination of feasibility. All patients had an inflammatory rheumatic disease, were mobile phone owners, capable of using text-message service on a mobile phone, and able to communicate in Norwegian.

### Comparison of SMS and P&P methods

Every day the patients reported pain, fatigue, stiffness and ability to carry out daily activities on four numerical rating scales (NRS) ranging from 0 – 100 (0 = no pain/fatigue/stiffness/my disease do not hinder daily activities, 100 = worst possible pain/fatigue/stiffness/my disease completely hinder daily activities). Every other day the four variables were reported with SMS and every other day with P&P for 28 consecutive days. In total each patient had to complete 14 questionnaires (P&P) and 14 text messages (SMS).

The SMS method has a 160 characters limit per message, thus the text on the questionnaires was abbreviated to the following text:

State degree of pain, fatigue, stiffness and how the disease affects your ability to conduct daily activities at present. 0 = best possible, 10 = worst possible. Remember full stop separating the numbers. Thank you!

**Figure 1:**
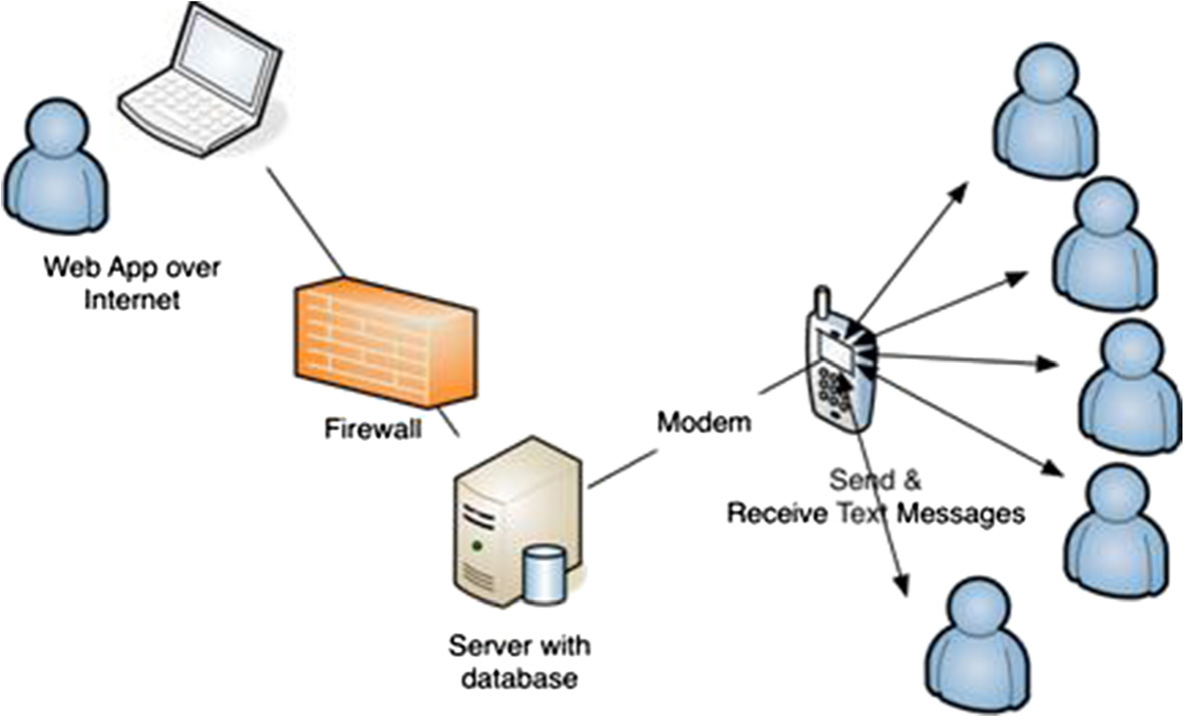
Overview of the SMS data collection system.

This text is 160 characters in Norwegian. Complete text, equal to the text on the questionnaires, was printed on small cards that the patients could carry with them. When receiving the SMS message, the patients scored each of the four variables and separated the scores with a period. A correct SMS response could for instance be: “1.5.7.3”. If no SMS response to the initial message was returned within 24 hours, it was recorded as missing. Data from the SMS were directly transferred electronically via the mobile phone network to a central, secure server. Through a secure website, the researcher could check the status of the responses and download the data for analysis into the statistical software package (Figure 1).

Completed P&P forms were put in postage prepaid envelopes and returned to the researcher at end of each week. Data from the paper forms were entered manually into the statistical software package (SPSS, version 20).

### Feasibility of the SMS method

In a clinical multicentre study, with three participating centres, we investigated the influence of exercising in heated water. Twice a week the four variables pain, fatigue, stiffness and ability to carry out daily activities were assessed with SMS for at total of 35 weeks. The text in the messages, procedures of reporting, collecting and transfer of data were equal to the SMS procedures described above, except that a reminder message was automatically sent to nonresponders after 24 hours by simply sending the same question a second time. If no response was recorded within the next 24 hours, the value was recorded as missing.

### Data analysis – comparison of SMS and P&P methods

Mean scores and variation, expressed as the pooled standard deviation (SD) of the mean score or as the mean ranges between the maximum and minimum scores for the two methods, were compared using paired sample t-tests or Wilcoxon Signed Rank Test. The level of statistical significance was set to p ≤ 0.05. If more than three text messages or P&P forms were missing, the patient was excluded from the analyses.

### Data analysis – feasibility of the SMS method

Feasibility of the SMS method was expressed as response-rate (%) in the total group and response-rate per centre. If more than five SMS were missing, the patient was excluded from the analyses.

### Ethical considerations

All participants signed an informed consent form and the study was approved by the Regional Ethics Committee for Medical Research in Norway (2011/1654).

## Results

### Comparison of SMS and P&P methods

#### ***Excluded group***

Twenty-eight patients were included for comparison of methods. Seven patients were excluded from the analysis because of more than three SMS and/or P&P forms missing. Five patients missed all P&P forms (14 forms), one patient missed 11 P&P forms, while three patients missed 8 SMS, 6 SMS and 4 SMS, respectively. Two patients missed > 3 responses in both formats.

Demographic data (age, sex, disease duration) were collected in the first questionnaire. Five of the patients in the excluded group did not return the first questionnaire. Thus comparisons between patients in the included group and patients in the excluded group were not performed as relevant information was only accessible for two of the patients in the excluded group.

#### ***Included group***

Twenty-one patients, mean (SD) age 49.7 (12.2) years and 76% (16 patients) female gender are included in the analyses. All 21 patients reported disease duration >1 year, of which 90% (18 patients) reported disease duration >6 years. All patients included in the analysis had three or less missing paper questionnaires or text messages.

There was no significant difference in mean scores reported with the SMS method compared to the mean scores reported with the P&P method (Table 1).

**Table 1:** Comparisons of mean scores, standard deviations of mean scores (SD) and mean range scores

	**Scores (mean)**			**SD (mean scores)**			**Range (mean)**		
**Variable (NRS)**^**a**^	**SMS**	**P&P**	**p-value**	**SMS**	**P&P**	**p-value**	**SMS**	**P&P**	**p-value**
Pain	3.45	3.47	0.76^b^	1.60	1.61	0.46^b^	3.33	3.43	0.55^c^
Fatique	3.66	3.64	0.92^b^	1.84	1.95	0.65^c^	3.71	3.52	0.55^b^
Stiffness	4.08	4.13	0.70^b^	1.84	2.03	0.16^b^	3.19	2.90	0.32^b^
Activity	3.29	3.32	0.84^b^	1.92	2.08	0.85^c^	3.90	3.67	0.37^c^

^a^NRS = Numeric Rating Scale, 0 - 10, 0 = best, ^b^paired t-test, ^c^Wilcoxon Signed Rank test.

The variations in the four variables, expressed as the pooled standard deviation of the mean scores (SD) or as the mean range scores were comparable between the two methods (Table 1).

#### ***Feasibility of the SMS method***

Five patients (12.2%) were excluded from the clinical multicentre study because of other serious diseases or long-lasting journeys abroad. Thus 36 patients; mean (SD) age of 53.8 (11.8) years, 60% female gender (21 patients) and 92% (33 patients) with disease duration > 6 years were included in the feasibility analyses.

In total 2508 SMS were sent out. Fifty-two of the SMS were not returned, resulting in a response rate of 97.9% in the total group. Each patient received around 70 SMS, 10 (27.8%) patients responded to all SMS, 24 (66.7%) patients missed ≤ 3 SMS, and none of the patients missed ≥ 5 SMS during the 35 week study period.

Response-rates at each of the centres were 97.1% (816 of 840 possible), 98.3% (971 of 988 possible) and 98.4% (669 of 680 possible), respectively. Sixteen (30.7%) of the missing responses were due to using a letter instead of a number (6 SMS), technical problems with the server (8 SMS) or reporting numbers outside the range of 0 – 10 (2 SMS). Two-hundred and thirty-eight (9.5%) reminder SMS were sent out, of which 210 (87.7%) were answered.

## Discussion

### Comparison of SMS and P&P methods

As far as we know, no other studies have compared patient reported outcomes assessed with mobile phones (SMS) and paper questionnaires (P&P) in patients with inflammatory rheumatic diseases. A review comparing whether computer-administered tests were equivalent to their P&P forms concluded that health scores obtained via the two modalities were directly comparable [7]. The review used a test-retest correlation as the standard of comparison. It might be argued that an average weighted correlation is a more appropriate measure of agreement between the two methods than the mean scores and their standard deviation as used in our study. However, as we have 14 repeated measurements within each method per patient, calculation of an average weighted correlation would tend to even out the differences.

When using a personal digital assistant or a computer it is possible to write more text than within a single SMS message. Because of the 160-character limit in the SMS format, we had to abbreviate the P&P questions. Our choice of presenting all four variables in one text message can be questioned. In a study evaluating feasibility of SMS for asthma diary data collection the patients complained of receiving a sequence of three SMS instead of just one [8]. Presenting more than one message implies a delay between the user’s reply to the message and the reception of the next message of approximately one minute. This is the time needed for the reply message to reach the server, the server to validate the reply and send out the next message and for the next message to reach the user [9]. We decided to use one message per communication in order to decrease the risk of non-compliance and patients dropping out.

When receiving the SMS message, the patients scored each of the four variables as a number code. In a study converting SMS answers into simple number codes, none of the participants reported any difficulty using the simple codes. In fact, the authors believed that simplifying the logistics was an important factor in achieving a high response rate [9].

To actually describe the individual course in a fluctuating condition such as inflammatory rheumatic diseases, data collection at regular and tight intervals are needed [10,11]. This requires real-time assessment and, as a consequence, methods of reporting that are not prone to retrospective data entries. It is well known that P&P registrations are not always filled in at the appropriate time [6,12,13]. Retrospective data entry has implications on memory retrieval of symptom occurrence and intensity; that is, fluctuations during the week might be missed and patients might report symptoms as perceived during the last days rather than on daily basis [13,14]. The risk of retrospective reporting calls into question the reliability and validity of the P&P method in data collection of PROs. In contrast, the time stamped data collected with the SMS method precludes retrospective reporting and falsification of the data with regard to real time.

### Feasibility of the SMS method

Further, the SMS method was highly feasible in a multicentre clinical study with frequent data collection of patient-reported outcomes.

We interpreted response-rates as a measure of feasibility and found a mean response-rate of 97.9% over a 35 week period. Two studies on patients with low back pain, another fluctuating condition, have reported response-rates of 63% and 82.5%, respectively [15,16]. The latter was a multicentre study where the patients were followed weekly for 6 months and 11% of the patients were telephoned personally for reminders [16]. In our study a reminder SMS was sent out to 9.5%. Our results indicate that patients with an inflammatory rheumatic disease were comfortable with the SMS method and that the method is highly feasible in a multicentre setting.

The fact that mobile phone ownership is high across the world and that people carry their phones with them almost all time make the mobile phones feasible tools for frequent data assessments [17]. Further, a high proportion of patients up to age 65 were successfully using SMS despite older age or functional disability caused by rheumatic diseases [18]. From the clinician and researcher’s point of view, the SMS method is more feasible than its P&P equivalent. The SMS method may lead to a reduction in data management burden, processing time and costs compared to the P&P data collection method [6-8,19].

### Limitations

A limitation of the SMS method is the 160-character limit per message. This affects the number of questions one can pose, as well as the amount of information the responses can contain. Further, patient mistakes during data entry are possible, i.e. using letters instead of numbers, not separating the numbers with a period, or sending responses outside the range; all mistakes that are interpreted by the server as a non-response. In our study we predefined responses outside the range of 0-10 as missing responses, while we accepted numbers separated by something other than the period, i.e. a space.

## Conclusion

The P&P collection method has an important place in clinical research. However, in studies requiring frequent data collection and real-time assessments, the SMS method may outperform the P&P method and provide high response rates. Questions suitable for SMS should be short and preferably answered in an equally short manner, thus the SMS method seems best suited for single-item instruments like the numeric rating scale.

## Competing interests

The authors declare that they have no competing interests.

## Authors’ contributions

All authors were involved in conception and design of the project. KBH largely originated the methodology. HD, AC, ØD and TS designed and implemented the electronic collection solution. AC and HD coordinated the data collection. AC, HD and KBH performed the analysis and wrote the first draft of the manuscript, including figures and tables. All authors contributed substantially to revisions of the manuscript, and read and approved the final version.

## Pre-publication history

The pre-publication history for this paper can be accessed here:

http://www.biomedcentral.com/1471-2288/14/52/prepub

## References

[B1] Heiberg T Kvien TK Dale O Mowinckel P Aanerud GJ Songe-Moller AB Uhlig T Hagen KB Daily health status registration (patient diary) in patients with rheumatoid arthritis: a comparison between personal digital assistant and paper-pencil format Arthritis Rheum 2007 57 3 454 460 10.1002/art.22613 17394232

[B2] Mowinckel P Hagen KB Heiberg T Kvien TK Repeated measures in rheumatoid arthritis reduced the required sample size in a two-armed clinical trial J Clin Epidemiol 2008 61 9 940 944 10.1016/j.jclinepi.2007.12.004 18538992

[B3] Felson DT Choosing a core set of disease activity measures for rheumatoid arthritis clinical trials J Rheumatol 1993 20 3 531 534 8478865

[B4] Kirwan JR Hewlett SE Heiberg T Hughes RA Carr M Hehir M Kvien T Minnock P Newman SP Quest EM Richards P Incorporating the patient perspective into outcome assessment in rheumatoid arthritis progress at OMERACT 7 J Rheumatol 2005 32 11 2250 2256 16265712

[B5] Kvien TK Mowinckel P Heiberg T Dammann KL Da le O Aanerud GJ Alme T Uhlig T Performance of health status measures with a pen based personal digital assistant Ann Rheum Dis 2005 64 10 1480 1484 10.1136/ard.2004.030437 15843456 PMC1755226

[B6] Lane SJ Heddle NM Arnold E Walker I A review of randomized controlled trials comparing the effectiveness of hand held computers with paper methods for data collection BMC Med Inform Decis Mak 2006 6 23 10.1186/1472-6947-6-23 16737535 PMC1513201

[B7] Gwaltney CJ Shields AL Shiffman S Equivalence of electronic and paper-and-pencil administration of patient-reported outcome measures: a meta-analytic review Value Health 2008 11 2 322 333 10.1111/j.1524-4733.2007.00231.x 18380645

[B8] Anhoj J Moldrup C Feasibility of collecting diary data from asthma patients through mobile phones and SMS (short message service): response rate analysis and focus group evaluation from a pilot study J Med Internet Res 2004 2 6(4) e42 15631966 10.2196/jmir.6.4.e42 PMC1550628

[B9] Kew S Text messaging: an innovative method of data collection in medical research BMC Res Notes 2010 3 342 10.1186/1756-0500-3-342 21172018 PMC3022815

[B10] Smolen JS Aletaha D Monitoring rheumatoid arthritis Curr Opin Rheumatol 2011 23 3 252 258 10.1097/BOR.0b013e328345743a 21427576

[B11] Sen D Brasington R Tight disease control in early RA Rheum Dis Clin North Am 2012 38 2 327 343 10.1016/j.rdc.2012.04.004 22819087

[B12] Bialocerkowski AE Grimmer KA Milanese SF Kumar VS Application of current research evidence to clinical physiotherapy practice J Allied Health 2004 33 4 230 237 15656253

[B13] Stone AA Shiffman S Schwartz JE Broderick JE Hufford MR Patient non-compliance with paper diaries BMJ 2002 18 324(7347) 1193 1194 12016186 10.1136/bmj.324.7347.1193 PMC111114

[B14] Lauritsen K Degl IA Hendel L Praest J Lytje MF Clemmensen-Rothne K Wiklund I Symptom recording in a randomised clinical trial: paper diaries vs electronic or telephone data capture Control Clin Trials 2004 25 6 585 597 10.1016/j.cct.2004.09.001 15588745

[B15] Kongsted A Leboeuf-Yde C The nordic back pain subpopulation program–individual patterns of low back pain established by means of text messaging: a longitudinal pilot study Chiropr Osteopat 2009 17 11 10.1186/1746-1340-17-11 19919715 PMC2781014

[B16] Axen I Bodin L Bergstrom G Halasz L Lange F Lovgren PW Rosenbaum A Leboeuf-Yde C Jensen I The use of weekly text messaging over 6 months was a feasible method for monitoring the clinical course of low back pain in patients seeking chiropractic care J Clin Epidemiol 2012 65 4 454 461 10.1016/j.jclinepi.2011.07.012 22169083

[B17] The International Telecommunication Union [http://www.itu.int/en/ITU-D/Statistics/Pages/default.aspx]

[B18] Hughes LD Done J Not 2 old 2 TXT There is potential to use email and SMS text message healthcare reminders for rheumatology patients up to 65 years old Health Inf J 2011 17 4 266 276 10.1177/1460458211422019 22193827

[B19] Dale O Hagen KB Despite technical problems personal digital assistants outperform pen and paper when collecting patient diary data J Clin Epidemiol 2007 60 1 8 17 10.1016/j.jclinepi.2006.04.005 17161749

